# Osmotic Demyelination Syndrome in an Alcohol-Dependent Patient With Alcohol-Related Peripheral Neuropathy: A Case Report

**DOI:** 10.7759/cureus.112757

**Published:** 2026-07-15

**Authors:** Hideya Itagaki, Tomoyuki Endo

**Affiliations:** 1 Department of Emergency and Disaster Medicine, Tohoku Medical and Pharmaceutical University Hospital, Sendai, JPN

**Keywords:** alcohol dependence, alcohol-related peripheral neuropathy, case report, hyponatremia, osmotic demyelination syndrome

## Abstract

Osmotic demyelination syndrome (ODS) is a rare neurological disorder typically associated with rapid correction of hyponatremia, whereas alcohol-related peripheral neuropathy (ALN) is a common complication of chronic alcohol use. The coexistence of ODS and ALN is uncommon and may obscure timely diagnosis. We report the case of a 51-year-old man with a long-standing history of alcohol dependence who presented to the emergency department with vomiting and diarrhea persisting for six days. Laboratory tests revealed hyponatremia, macrocytic anemia, thrombocytopenia, and electrolyte imbalances. Neurological examination demonstrated distal paresthesia, diminished reflexes in the lower extremities, and positive Babinski and Chaddock signs. MRI revealed a hyperintense trident-shaped lesion in the pons consistent with ODS. These findings suggested the concomitant presence of ALN and ODS. The patient was treated conservatively with vitamin supplementation, careful correction of electrolytes, and gradual nutritional rehabilitation. Sodium levels normalized without the need for rapid correction, and the patient's symptoms improved, leading to discharge on the eighth hospital day. This case underscores two essential points: ODS was identified before any documented rapid in-hospital correction of hyponatraemia, and alcohol-dependent patients may present with overlapping features of ALN and ODS. Recognition of pyramidal signs is critical for differentiating central from peripheral neurological complications in this population. Clinicians should maintain a high index of suspicion for concomitant ODS in alcohol-dependent patients with neuropathy, particularly when pyramidal signs are present.

## Introduction

Alcohol-related peripheral neuropathy (ALN) is a common neurological complication associated with chronic alcohol consumption, with a prevalence rate of approximately 40% among long-term drinkers [1]. It typically presents as a progressive, length-dependent sensory neuropathy characterized by distal paresthesia, sensory loss, and reduced reflexes [2].

Chronic alcohol use may affect both the peripheral and central nervous systems. Osmotic demyelination syndrome (ODS) is a rare neurological disorder characterized by non-inflammatory demyelination in response to osmotic stress. Although ODS has historically been associated with rapid correction of hyponatremia, recent evidence suggests that the relationship between the rate of sodium correction and the development of ODS is more complex than previously recognized [3,4]. Additional factors associated with an increased risk of ODS include chronic alcohol use, malnutrition, liver disease, severe hyponatremia, and hypokalemia. ODS may present with various neurological manifestations, including dysphagia, dysarthria, altered mental status, and quadriparesis [5].

Here, we report a middle-aged man with alcohol dependence who presented with ALN and was found to have ODS before any documented rapid in-hospital correction of hyponatremia. This case highlights the importance of considering coexisting peripheral and central neurological disorders in patients with chronic alcohol use, particularly when neurological findings cannot be fully explained by peripheral neuropathy alone.

## Case presentation

A 51-year-old man with a history of alcohol use disorder, hyperlipidemia, vertebral fracture, and right shoulder fracture presented to the emergency department with vomiting and diarrhea that had persisted for six days. Several weeks before the presentation, the patient developed a loss of appetite and was unable to maintain adequate food intake, although he remained able to drink fluids and continued to consume alcohol, which constituted most of his oral intake. At baseline, he drank approximately three glasses of shochu (Japanese distilled spirit) mixed with water per day, typically beginning in the morning, and a 1.8-L bottle usually lasted four to five days. After his appetite decreased, his alcohol consumption also appeared to decrease, although the exact amount was not documented. His social history revealed a smoking habit of 15 cigarettes per day for 31 years (approximately 23 pack-years). Six days prior to presentation, he developed persistent hiccups followed by vomiting and watery diarrhea. As his condition did not improve, he called for emergency assistance and was admitted to our hospital.

On arrival, his level of consciousness was Japan Coma Scale 1 [6,7] (Glasgow Coma Scale E4V5M6). Vital signs were blood pressure 148/104 mmHg, heart rate 111 beats per minute, peripheral oxygen saturation (SpO₂) 100% (room air), and temperature 36.9°C. Physical examination revealed mild scleral icterus, mild conjunctival pallor, a surgical scar on the right shoulder, a flat and soft abdomen without tenderness but with collateral circulation, and no peripheral edema. Initial neurological examination revealed distal paresthesia in both lower extremities, with no apparent abnormalities in the upper extremities or fingers and no evident motor deficits. Detailed assessment of gait, proprioception, vibration sensation, and pinprick sensation was not documented at the initial examination. Point-of-care ultrasonography showed a negative sonographic Murphy sign.

Venous blood gas analysis showed mild respiratory alkalosis. Laboratory testing revealed hyponatremia (serum sodium, 125 mmol/L), hypophosphatemia, hypomagnesemia, elevated liver enzymes and lipase levels, and macrocytosis (Table 1).

**Table 1:** Initial laboratory findings at presentation ALP, alkaline phosphatase; ALT, alanine aminotransferase; APTT, activated partial thromboplastin time; AST, aspartate aminotransferase; BS, blood sugar; BUN, blood urea nitrogen; Ca, calcium; Cl, chloride; CRP, C-reactive protein; D-bil, direct bilirubin; Hb, hemoglobin; HCO₃⁻, bicarbonate; Hct, hematocrit; K, potassium; LDH, lactate dehydrogenase; MCH, mean corpuscular hemoglobin; MCV, mean corpuscular volume; Mg, magnesium; Na, sodium; P, phosphorus; pCO₂, partial pressure of carbon dioxide; PCT, procalcitonin; PT-INR, prothrombin time–international normalized ratio; RBC, red blood cell count; T-bil, total bilirubin; TP, total protein; VWBC, white blood cell count; γGT, gamma-glutamyl transpeptidase

Parameters	Patient Values	Reference Ranges
Complete blood count		
WBC	5.4	3.3~8.6（×10^3/μL）
RBC	3.57	4.35~5.55（×10^6/μL）
Hb	13	13.7~16.8（g/dL）
Hct	36.5	40.7~50.1（%）
Platelet count	45	158~348（×10^3/μL）
MCV	102.2	83.6~98.2（fL）
MCH	36.5	30.5~34.2（pg）
Fibrinogen	253	200~400（mg/dL）
APTT	25.8	24~39（sec）
PT-INR	0.89	0.90～1.15
D-dimer	3.09	0~1.0（μg/mL）
Biochemistry Data		
TP	7.3	6.6~8.1（g/dL）
Albumin	4.5	4.1~5.1（g/dL）
T-bil	3.9	0.4~1.5（mg/dL）
D-bil	2.09	0.05~0.30（mg/dL）
AST	34	13~30（U/L）
ALT	20	10~42（U/L）
ALP	65	38~113（U/L）
γGT	133	13~64（U/L）
LDH	137	124~222（U/L）
Lipase	310	13~55（U/L）
BUN	20	8~20（mg/dL）
Creatinine	0.79	0.65~1.07（mg/dL）
Na	125	138~145（mmol/L）
K	3.8	3.6~4.8（mmol/L）
Cl	86	101~108（mmol/L）
Ca	10.1	8.8~10.1（mg/dL）
P	0.9	2.7~4.6（mg/dL）
Mg	1.1	1.6~2.6（mg/dL）
BS	110	70-110（mg/dL）
Vitamin B1	15	21~82 ng/mL
Vitamin B2	55.5	66.１~111.4 ng/mL
Vitamin B12	430	197~771 pg/mL
Folic acid	22	4〜（ng/mL）
CRP	1.15	0.0~0.14（mg/dL）
PCT	0.12	0.0~0.05（ng/mL）
Venous blood gas		
pH	7.457	7.35–7.45
pCO₂	31.8	35–45 (mmHg)
HCO₃⁻	22.2	22–26 (mmol/L)
Lactate	1.9	<2.0 (mmol/L)

**Figure 1:**
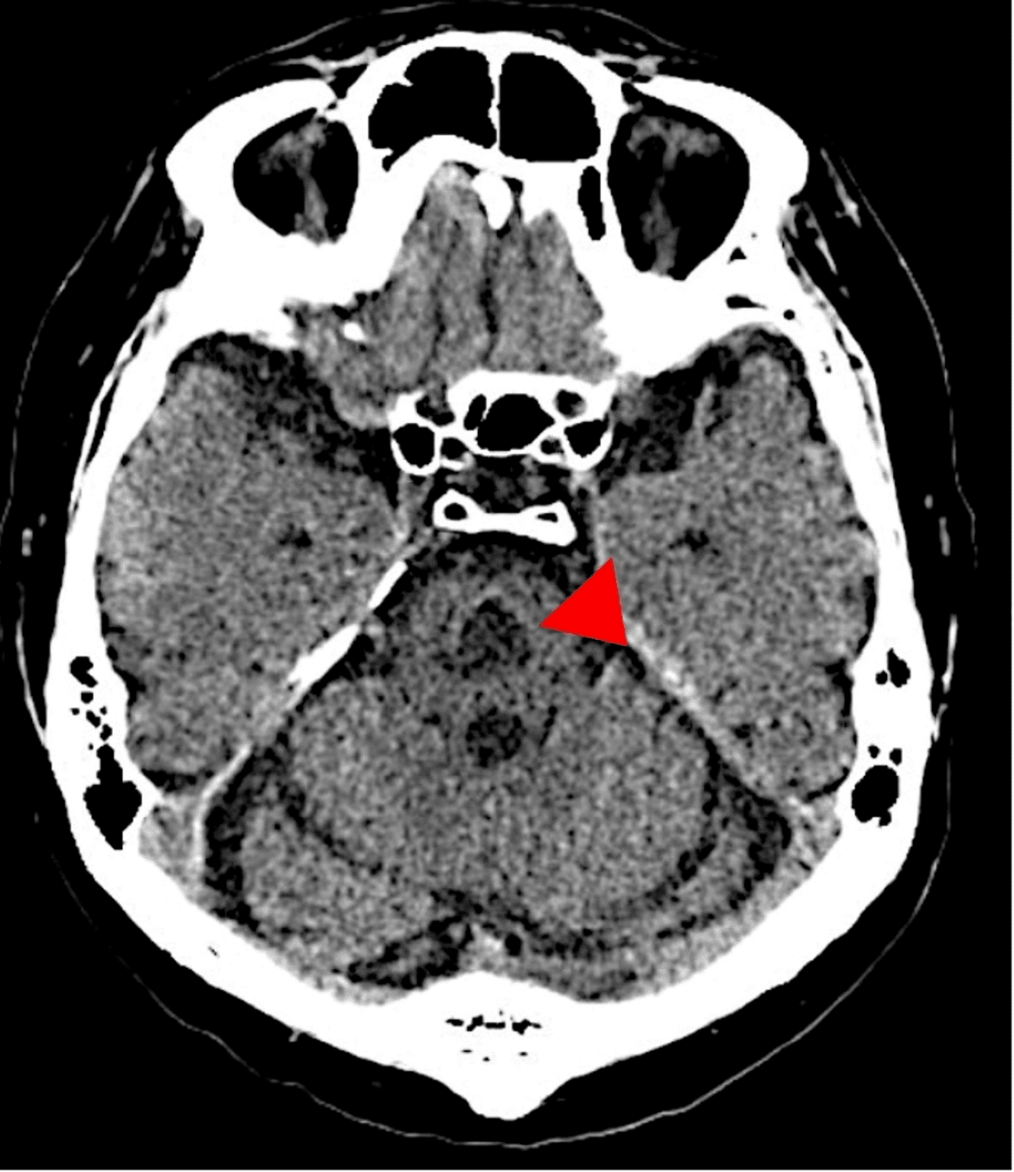
Axial non-contrast CT showing a hypodense lesion in the central pons (red arrowhead), consistent with ODS ODS, osmotic demyelination syndrome

Non-contrast CT of the head revealed a low-density lesion in the central pons (Figure 1). Contrast-enhanced CT of the trunk was obtained to investigate a possible underlying cause of suspected syndrome of inappropriate antidiuretic hormone secretion (SIADH) and showed no significant abnormalities.

**Figure 2:**
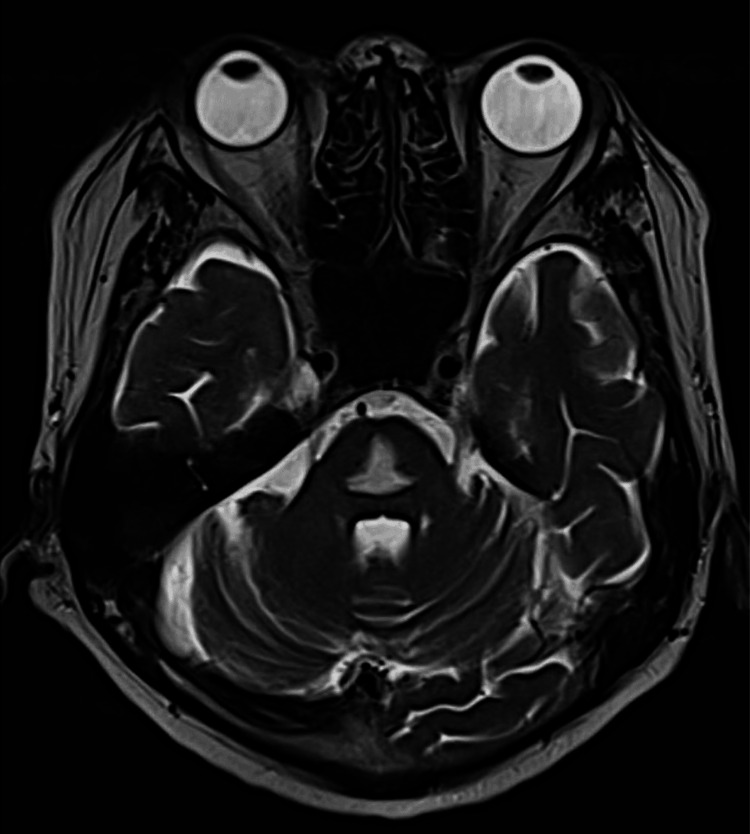
Axial T2-weighted MRI of the brain showing a symmetric hyperintense lesion in the central pons with a characteristic trident-shaped appearance, consistent with ODS ODS, osmotic demyelination syndrome

Brain MRI was subsequently obtained, which revealed a central pontine lesion that was hyperintense on T2-weighted images, hypointense on T1-weighted images, and mildly hyperintense on fluid-attenuated inversion recovery (FLAIR) images, without diffusion restriction on diffusion-weighted imaging. The imaging findings were considered suggestive of ODS (Figure 2).

Based on these imaging findings, a neurology consultation was obtained. The patient was alert and oriented, with no cranial nerve deficits or limb paralysis. He reported abnormal paresthesia, described as a “tingling sensation,” in both distal ankles. Deep tendon reflexes were diminished in the lower extremities but preserved in the upper extremities, suggesting peripheral neuropathy. Given his chronic alcohol use, prolonged inadequate oral intake, and deficiencies of vitamins B1 and B2, both alcohol-related and nutritional neuropathies were considered. However, nerve-conduction studies and electromyography were not performed. In contrast, bilateral Babinski and Chaddock signs were positive, indicating pyramidal tract involvement. Together with the characteristic central pontine lesion on MRI, these upper motor neuron signs supported concomitant central nervous system involvement due to ODS. The patient did not have dysphagia, dysarthria, cranial nerve deficits, altered consciousness, or limb weakness. The neurologist considered that no specific neurological intervention was required and that the patient’s nausea was unlikely to be directly attributable to ODS.

Accordingly, management focused on cautious correction of hyponatremia, vitamin replacement, nutritional rehabilitation, and prevention of refeeding complications. Oral intake was gradually advanced, and intravenous vitamin supplementation was administered. Serum sodium increased from 125 mmol/L at admission to 127 mmol/L the following day, 129 mmol/L on hospital day 3, and 138 mmol/L on hospital day 6. Because serum sodium was not measured daily between hospital days 3 and 6, the maximum 24-hour correction rate during this interval could not be determined. He resumed normal oral intake and was discharged on hospital day 8. His nausea resolved, but the distal paresthesia, diminished lower-extremity reflexes, and bilateral Babinski and Chaddock signs persisted at discharge.

## Discussion

Two key lessons were learned from this case. First, the patient had already developed ODS at the time of presentation with hyponatremia, even though sodium had not been rapidly corrected. Second, in patients with alcohol use disorder, ODS may coexist with ALN.

To better understand these findings, it is essential to review the historical and pathophysiological background of ODS. ODS was first described in 1959 by Adams et al. as a neurological disorder characterized by non-inflammatory demyelination of the central pons [8]. In 1982, Norenberg et al. analyzed 12 autopsy cases of ODS and found that serum sodium levels had risen by more than 20 mmol/L within three days. This led to the widespread recognition that rapid sodium correction is a significant trigger of ODS [9].

After adaptation to chronic hyponatremia, the brain becomes vulnerable to osmotic stress during correction [10]. Therefore, during rapid correction of sodium, intracellular electrolyte concentrations recover rapidly, whereas organic osmolytes recover more slowly. This disequilibrium is believed to cause cellular dehydration, which, in turn, leads to myelin damage and oligodendrocyte degeneration [11]. However, subsequent reports have indicated that factors other than sodium correction may also contribute to the development of ODS. These include hyperglycemia, folate deficiency, liver cirrhosis, and, as in the present case, alcohol use disorder [11-14]. 

Several pathophysiological mechanisms have been proposed to explain alcohol-related ODS. Patients with alcohol use disorder are often deficient in organic osmolytes, making their cells, particularly oligodendrocytes, more susceptible to shrinkage and demyelination [15]. In addition, malnutrition, which frequently accompanies alcohol dependence, predisposes to inadequate energy reserves needed to maintain Na⁺/K⁺-ATPase pump activity and to synthesize organic osmolytes [11,15].

Furthermore, thiamine deficiency may impair cerebral glucose uptake, thereby further exacerbating the disease process [11].In this case, several predisposing factors, including alcohol dependence, prolonged inadequate oral intake, hyponatremia, and deficiencies of vitamins B1 and B2, may have increased the patient’s susceptibility to ODS. Recent evidence has challenged the traditional view that the rate of sodium correction is the principal determinant of ODS. In a systematic review and meta-analysis of hospitalized adults with severe hyponatremia, more rapid correction was not associated with a statistically significant increase in ODS, whereas slower correction was associated with higher mortality and a longer hospital stay [16]. These findings suggest that patient-related vulnerability, including alcohol dependence, malnutrition, and other metabolic abnormalities, may be important in the pathogenesis of ODS. The combination of these factors may therefore have contributed to the development of ODS in the present patient.

Clinical manifestations of ODS comprise dysphagia, dysarthria, spastic quadriparesis, pseudobulbar palsy, ataxia, lethargy, tremors, and disorientation. In severe cases, the condition may progress to locked-in syndrome or coma [5]. Although the prevalence of Babinski and Chaddock signs in ODS has not been precisely quantified, these signs result from pyramidal tract involvement and have been documented in case series [17]. In contrast, clinical features of ALN comprise distal sensory disturbances with a glove-and-stocking distribution, manifesting as pain, paresthesia, and sensory loss, along with distal muscle weakness and atrophy (predominantly in the lower limbs), loss of deep tendon reflexes, and autonomic fiber involvement [2]. Because ALN is a peripheral neuropathy, Babinski and Chaddock signs are not expected to be present. Therefore, the presence of these pyramidal signs in a patient with alcohol use disorder should raise suspicion of concomitant ODS.

No disease-specific treatment for ODS has been established, and management is primarily supportive [3]. In contrast, management of ALN focuses on alcohol abstinence and correction of nutritional deficiencies, although evidence regarding specific vitamin therapy remains limited [1]. Recognizing the coexistence of ODS and ALN is of considerable clinical importance, as their overlapping features may obscure the diagnosis. A careful neurological examination, particularly the detection of pyramidal signs such as the Babinski and Chaddock responses, can help distinguish central involvement due to ODS from peripheral neuropathy due to ALN. Our case highlights the importance of careful clinical evaluation, as ODS was identified at presentation before any documented rapid in-hospital correction of hyponatremia.

## Conclusions

ODS can develop in alcohol-dependent patients even in the absence of rapid sodium correction. The presence of pyramidal signs, such as Babinski and Chaddock responses, should raise suspicion of concomitant ODS in patients with ALN. Further studies are warranted to clarify the prevalence, risk factors, and outcomes of ODS in this specific patient population. This case emphasizes the importance of considering both central and peripheral neurological complications in alcohol-dependent patients.
